# Brain regions beyond the visual cortex are relevant to subjective time prediction from fMRI salient events in a visual naturalistic context

**DOI:** 10.1007/s11682-026-01128-8

**Published:** 2026-03-21

**Authors:** Erick Almeida de Souza, Carlos Ernesto Garrido Salmon

**Affiliations:** 1 InBrain, Department of Physics, FFCLRP, University of São Paulo, Ribeirão Preto, Brazil; 2 Department of Medical Imaging, Hematology and Clinical Oncology, FMRP, University of São Paulo, Ribeirão Preto, Brazil

**Keywords:** Human time perception, fMRI, Machine Learning, Cerebral cortex

## Abstract

Time perception is an essential part of human experience and can be distorted by features of daily stimuli, causing variations relative to actual time. This study aimed to test the hypothesis that subjective time results from the accumulation of salient events and investigate the neural bases of this perceptual processing, while using a publicly available dataset obtained and analyzed in a previous study. Three approaches were used with fMRI data from participants who watched and estimated the duration of silent videos in an MRI scanner. In the first approach, using a functional parcellation of 360 cortical regions, we showed that salient events in functional networks beyond the visual one also had a significant association with subjective time (normalized bias): the dorsal attention network, the cingulo-opercular network, and the somatomotor network. In the second approach, using an Long-Short Term Memory (LSTM) deep learning model, it was not possible to predict normalized bias from fMRI trial-by-trial time series within any functional network. In the last approach, functional connectivity between the 360 cortical regions and within the visual network showed no consistent association with subjective time. The results suggest that regions in additional functional networks may be important for duration quantification in the context of visual stimulation, although the approach based on salient events still requires further validation. Furthermore, features such as functional connectivity and fMRI time-series did not show direct association with the normalized bias.

**Supplementary Information:**

The online version contains supplementary material available at 10.1007/s11682-026-01128-8.

## Introduction

The human brain is able to process different time-scales (microseconds, milliseconds, seconds and circadian timing) using different neural systems and mechanisms (Buhusi & Meck, 2005; Allman et al., 2014; Merchant & Lafuente, 2014; Paton & Buonomano, 2018). The conscious temporal processing in the seconds to minutes range is called interval timing.

In the recent literature, researchers have been focusing their attention to develop intrinsic rather than dedicated timing models (Fountas et al., 2022; Jong, 2024), since there is no consistent evidence in the literature that points to the existence of a dedicated system for time perception in the human brain and due to the flexibility of intrinsic models across stimulus modalities (Ivry & Schlerf, 2008; Coull et al., 2011; Merchant et al., 2013). The predictive processing model is an intrinsic model of time perception that relates episodic memory to time perception and it was recently developed by Fountas et al. (2022). According to this model, time is estimated by counting the number of surprising events encoded in a sensory network. In this framework, sensory processing networks are assumed to act as hierarchical Bayesian models (Friston & Kiebel, 2009): an internal model of the world is continually updated by comparing model-based predictions to novel sensory input.

Recent meta-analytical studies have investigated the brain structures associated with temporal processing across different neuroimaging experimental modalities, including variable stimulus duration scales and sensory categories (Nani et al., 2019; Naghibi et al., 2024; Li et al., 2023). The brain areas and structures more often pointed by these studies are the pre-supplementary motor area (pre-SMA), insula, inferior frontal gyrus (IFG), inferior parietal lobule (IPL), dorsal striatum (DST), and cerebellum. More specifically, the middle-frontal gyrus (MFG) and the IPL were often associated with visual and single-interval stimuli.

Some studies have explored the association and role of functional connectivity (van den Heuvel & Pol, 2010) in temporal processing (Carvalho et al., 2016; Grahn & Rowe, 2009). Stevens et al. (2007) applied Independent Component Analysis (ICA) to investigate this association and observed groups of functionally interconnected brain regions that were activated across different auditory-related interval timing tasks. Particularly, they highlighted the prominence of a frontostriatal timing neural network. In an fMRI experiment involving visual and auditory modalities, Liu et al., 2023 showed that the functional connectivity between the left caudate and left precuneus likely plays an important role in time estimation in a time reproduction task compared to a control task. Teghil et al. (2020) have recently investigated the association between inter-subject variations in resting-state functional connectivity and the individual performances in an auditory-related time reproduction task. The authors found an increased resting-state connectivity between the right insular cortex and precentral gyrus and between the precentral gyrus and the ipsilateral putamen that can predict individual values of interval timing accuracy.

No dedicated or intrinsic timing theory was consistently corroborated by experimental investigation so far. Sherman et al. (2022) has recently tested the hypothesis that *“differences from clock time in subjective experience of time arise because time estimates are constructed by accumulating the same quantity that guides perception: salient events”* (Sherman et al., 2022). The authors recruited several subjects to watch and estimate the duration of two categories of silent videos during a fMRI acquisition, which differ in the amount of events and changes during the video span. The neural change in brain processing was defined as the signed difference between consecutive timepoints in the BOLD time-series and each change value was classified as salient or not following a criterion function. Finally, they evaluated the association between salient events within a visual hierarchical brain parcellation and a behavioral measure of subjective time bias as compared to control models of auditory and somatosensory hierarchies. The authors reported that a significant association was observed only for the visual perceptual hierarchy, as expected, since the experiment was based on visual stimulus.

In the current work, we aim to validate the hypothesis proposed by Sherman et al. (2022) using their data. It was suggested in Sherman et al. (2022) that since the presented stimuli were silent videos, only the salient events accumulated in the visual hierarchical cortex would be associated with subjective time. In Approach 1, by using an alternative brain parcellation scheme while keeping the rest of the methodology unmodified, we hypothesize that different regions or networks could play important roles in time perception as well. In Approach 2, we evaluate whether a Long-Short Term Memory (LSTM) model is able to automatically capture salient changes in the time-series of fMRI data without the need of a predefined criterion function. Approaches 2 and 3 are therefore exploratory in nature and were not designed to directly validate the salient-event hypothesis, but rather to investigate complementary and data-driven associations between brain dynamics and subjective time. Finally, in Approach 3, we investigated whether brain functional connectivity would be associated with subjective time, more specifically, with normalized duration bias. An important motivation for this approach were the results obtained in Approach 1: salient events captured from different brain functional networks showed positive associations to normalized bias.

## Methodology

### Experimental data and pre-processing

In this work, we performed a cross-sectional, retrospective study involving young, healthy individuals. We used the dataset analyzed in Sherman et al. (2022), which is publicly available on the OSF repository (https://osf.io/2zqfu*).* The dataset includes 40 right-handed, English-speaking healthy participants, ranging from 18 to 43 years old, with 26 women and 14 men. However, for the following analyses, image data from two participants were removed due to corruption. Therefore, the final dataset included fMRI data from 38 subjects.

The MRI technique employed was functional T2*-weighted multi-band echo-planar imaging (EPI). Images were captured using a Siemens PRISMA 3T scanner with a 64-channel head coil, under the following conditions: 72 axial slices, slice thickness = 2 mm, gap size = 2 mm, TR = 800ms, multiband factor = 8, TE = 37ms, and flip angle = 52°. Further details regarding auxiliary acquisitions (such as structural scans and field maps) can be found in the original paper by Sherman et al. (2022). Preprocessing was performed with the SPM12 software (http://www.fil.ion.ucl.ac.uk/spm/software/spm12*)*, and the key steps of the pipeline were as follows: functional and structural images were reoriented to the anterior commissure, EPIs were aligned to one another, corrected for distortions using field maps, and co-registered to the structural images. For approaches 2 and 3, an additional preprocessing step was applied for improving the signal-to-noise ratio: spatial smoothing with a Gaussian kernel of 4 mm FWHM.

Each participant’s experimental session lasted 60 min. During image acquisition, they watched silent videos of varying duration (8–24 s) on a computer screen positioned above the 64-channel head coil. The videos depicted either office or city scenes. For each duration and content condition, two videos were presented per block in a randomized order. After each video, participants estimated the perceived duration using a 0–40 s visual scale. There was no task training prior to the experiment session. Further details related to data acquisition can be accessed directly in the Sherman et al. (2022) article. In this experiment, a trial was defined as the video presentation period; all neural analyses were therefore restricted to this interval, while the response phase was excluded and used solely to obtain behavioral duration estimates.

### Behavioral metrics

The subjective time metrics evaluated in this work were the reported duration (x) and the.

normalized bias (Eq. 1). Normalized bias represents the participant’s bias in underestimating or overestimating the duration of videos in each category in relation to participant average report. For each subject, it is calculated for each trial k as the normalized difference between each estimated duration and the participant’s average estimate for the duration category t.

1 $$bias_{tk}=\frac{x_{tk}-\overline{x_t}}{\overline{x_t}}$$

### Brain parcellation

We applied a functional segmentation scheme to the pre-processed EPI files of each participant. We used the multi-modal cortical atlas (Glasser et al., 2016) with 360 regions (180 for each hemisphere). The atlas was obtained in MNI standard space and aligned with the pre-processed EPI data. Although both datasets were defined in the same standard pace, they differed in spatial resolution and dimensions. Therefore, the atlas was resampled using nearest-neighbor interpolation to match the EPI resolution, preserving discrete regional labels. Minor spatial adjustments were then performed to ensure an accurate overlap with the functional brain volume. This procedure was applied independently to each large-scale functional network prior to time-series extraction. It takes into consideration variations in architecture, function, connectivity, and/or topography throughout the human cortex. The main motivation for using this atlas is that the majority of brain regions and structures previously identified as being associated with interval timing are located in the cerebral cortex (Naghibi et al., 2024; Nani et al., 2019). Each region in the atlas can be classified into 14 functional networks, as described in Ito et al. (2017). In the present study, however, we focused on the seven largest canonical networks, which are more consistently identified across individuals and widely used in the fMRI literature (Beckmann et al., 2005; Dubois et al., 2018; Souza et al., 2023).

Through this approach, we aimed to assess whether certain regions or networks are particularly prominent compared to others in predicting normalized bias.

### Approach 1: prediction of normalized bias from salient events

We calculated BOLD signal change for each functional network and time-point (TP) as described in Eq. 2. First, we calculated the difference between the BOLD signal (X) at a given time TP for a voxel *v* and the activation at time TP − 1 for the same voxel; then, the difference is summed over the voxels of the network for the same TP. We opted to use the change relationship shown in Eq. 2 because the best results in Sherman et al. (2022) were achieved using this approach.

2 $${\varDelta}_{TP}={\sum}_{v}^{}\left({X}_{TP,v}-{X}_{TP-1,v}\right)$$

Changes were classified as salient or not by comparing each Δ_TP_ value to the dynamic criterion function ${\vartheta}_{TP}$ corrupted with Gaussian noise ε (Eq. 3). In this equation, 𝜗_min_ and 𝜗_max_ represent the standard deviations below and above the mean, respectively, for the z-scored Δ_TP_ values and N(0, 0.05) represents a normal distribution with a mean of 0 and a variance of 0.05. In the initial analyses, we used 𝜗_min_ = -1 and 𝜗_max_ = 1.5, the same values used for layer 1 in Sherman et al. (2022). The criterion function was reset to its initial value after the detection of each salient event.

3 $$\begin{array}{c}\vartheta_{TP}=\left(\vartheta_{max}+\vartheta_{min}\right)\times e^{-TP}+\vartheta_{min}+\varepsilon,\varepsilon\;N\left(0,0.05\right)\end{array}$$

To predict video duration values from salient changes, a Support Vector Regression (SVR) model was applied, in which the input is the number of salient events accumulated for each functional network, participant and trial. The model was implemented in Python using the sklearn package (Pedregosa et al., 2011). Using the predicted duration values, we calculated the respective model bias values following the same procedure applied to participants (Eq. 1), but using the SVR-derived predictions, and compared them with the observed bias values for each participant. We used a 10-fold cross-validation.

We evaluated the effect of the type of video (scene) on model normalized bias in each functional network. A linear mixed model (Eq. 5) was built using the scene as fixed effect and was compared with the reduced model (without the fixed effect) using a chi-squared test.

5 $$bia{s}_{model}\sim1+scene+\left(1\vee\:participant\right)$$

Robustness analysis was conducted by recalculating salient events with variations in the criterion parameters: ϑ_min_ was assigned 50 linearly spaced values between 3 SD and 0 SD below the mean, while ϑmax independently took 50 linearly spaced values between 0 SD and 2.5 SD above the mean. These parameters correspond to those used for the first hierarchical layer of the study by Sherman et al. (2022). Using these criteria, we generated 2,500 datasets for each ROI. For each ROI and dataset, we tested the association between model-predicted bias and human bias by fitting the regression model:

6 $$bia{s}_{human}\sim{\beta}_{0}+{\beta}_{1}\times\:bia{s}_{model}$$

Heat maps depicted in Fig. 2 correspond to one-tailed p-values for β_1_.

### Approach 2: prediction of normalized bias from bold time-series

In this approach, we sought to explore whether temporal processing information based on normalized bias or video duration values can be directly captured from BOLD time-series using a deep recurrent LSTM model (Hochreiter; & Schmidhuber, 1997). We chose to use this model due to its established ability to capture both long- and short-term dependencies in time-series data (Yu et al., 2019; Fan et al., 2020; Vieira et al., 2021). In this setup, each sample in the input matrix represented the BOLD time-series for each trial, with each trial’s time-series corresponding solely to the video-watching interval. The output was a vector containing the normalized bias or video duration values for each sample. To prevent bias in the results due to using data from the same subject in both training and testing samples, we implemented a leave-one-subject-out cross-validation approach. The LSTM algorithm was executed using the PyTorch package (Paszke et al., 2017). The input data consisted of time-series samples with variable lengths. For batch-wise processing with the LSTM, shorter sequences were zero-padded to match the length of the longest trial. The true sequence lengths were provided to the model, ensuring that padded time points were ignored during the LSTM computations. This resulted in an input matrix of size 2211 samples × number of regions × 31 time points, where zero padding served only as a technical placeholder.

The LSTM is composed of an input gate, an output gate, a forget gate and a cell (Hochreiter & Schmidhuber, 1997). The LSTM uses gated mechanisms to control information flow over time: the forget gate regulates memory retention, the input gate controls the incorporation of new information into the cell state, and the output gate determines how much of the internal state is exposed as the hidden representation. This design enables stable learning of long-range temporal dependencies. The following equations describe each gate/state:

7 $$Forget\;gate:\;f_t\;=\mathrm{Sigmoid}\;(W_fx_t+U_fh_{t-1}+bf)$$

8 $$Input\;gate:\;i_t=\mathrm{Sigmoid}(\;W_ix_t+U_ih_{t-1}+b_i)$$

9 $$Output\;gate:\;o_t=\mathrm{Sigmoid}\;(W_ox_t+U_oh_{t-1}+b_o)$$

10 $$\begin{array}{c}Estimated\;current\;cell-state:{\dot C}_t=\tanh\;(W_cx_t+U_ch_{t-1}+b_c)\end{array}$$

11 $$Cell-state:\;C_t=i_t\odot\dot C_t\;+\;f_t\odot C_{t-1}$$

12 $$Hidden-state:\;h_t=o_t\odot tanh(C_t)$$

Where W_f​_, W_i​_, W_o_​, and W_c_​ are the input weights; U_f_, U_i​_, U_o​_, and U_c_ are the recurrent weights; b_f_, b_i_, b_o_, and b_c_ are the bias terms; and ⊙ denotes the Hadamard product.

To enable the model to learn more complex dynamic features of the input data, we chose to employ two LSTM layers instead of just one. We averaged the outputs from the LSTM layers to integrate all fMRI steps. Finally, the learned features *h* were processed through a fully connected layer (Eq. 13). The cost function used was the Mean Squared Error (MSE).

13 $$\mathrm h^1=\mathrm b^1+\mathrm h^{1-1}\times\mathrm w^1$$

Where h^l^ denotes the output of layer *l*, computed as a linear transformation of the input from the previous layer h^l−1^ using the weight parameter w^l^, followed by the addition of a bias term b^l^.

We optimized the following hyperparameters for each training condition: learning rate, scheduler step size and scheduler gamma ratio. The scheduler was used to gradually decrease the learning rate over the training epochs.

### Approach 3: prediction of normalized bias from functional connectivity

To predict normalized bias and video duration from functional connectivity, we implemented an Elastic-net regression (Zou; & Hastie, 2005) using the sklearn package in Python. Elastic-net optimizes the model by estimating the β coefficients that minimize the cost function specified in Eq. 16. Lasso (Eq. 14) is particularly effective for selecting variables within a highly correlated group, as it reduces some coefficients to zero. Ridge (Eq. 15) regression also shrinks coefficients but retains all variables in the model. The balance between Lasso and Ridge penalties is regulated by the α hyperparameter, while the λ hyperparameter determines the weight of the overall Elastic-net regularization. This model has been successfully applied to fMRI functional connectivity data in previous works (Dubois et al., 2018; Souza et al., 2023; Teipel et al., 2017).

14 $${\lVert\beta\lVert}_{1}={\sum}_{j=1}^{p}{\left|\beta\right|}_{j}$$

15 $${\lVert\beta\lVert}_{2}=\sqrt{{\sum}_{j=1}^{p}{{\left|\beta\right|}_{j}}^{2}}$$

16 $$\begin{array}{c}\widehat\beta=argmin_\beta\left\{\frac1{2N}\sum\nolimits_{i=1}^N\left(y_i-{x_i^\top\beta}^{}\right)^2+\lambda\left(\alpha{\left|\left|\beta\right|\right|}_1+\frac12\left(1-\alpha\right){\left|\left|\beta\right|\right|}_2^2\right)\right\}\end{array}$$

The brain parcellation used in this approach was the 360-region functional atlas (Glasser et al., 2016) for the same motivation described in the previous approach. In this setup, each sample was the upper triangular functional connectivity matrix calculated as the Pearson correlation between each regional time-series of the parcellation for each trial of each subject. We optimized α and λ parameters within a range of 10 uniformly spaced values (Eq. 16). Similar to the previous approach, we trained the model using functional connectivity data extracted from two anatomical frameworks: (1) whole-brain data from Glasser’s 360 regions and (2) data from only 56 regions focusing on the visual network. This preparation yielded an input matrix with dimensions [2211 samples, number of regions, 64620 edges]. We used leave-one-subject-out cross-validation.

Alternatively, we also attempted to implement the strategy used in Sherman et al. (2022) of predicting video duration values and then computing the respective model’s normalized bias for each trial. We employed the Elastic-net model to predict video duration values based on the functional connectivity calculated within the visual network. We then computed the model’s normalized bias and assessed the association using linear regression and linear mixed models.

## Results

### Approach 1: prediction of normalized bias from salient events

**Fig. 1:**
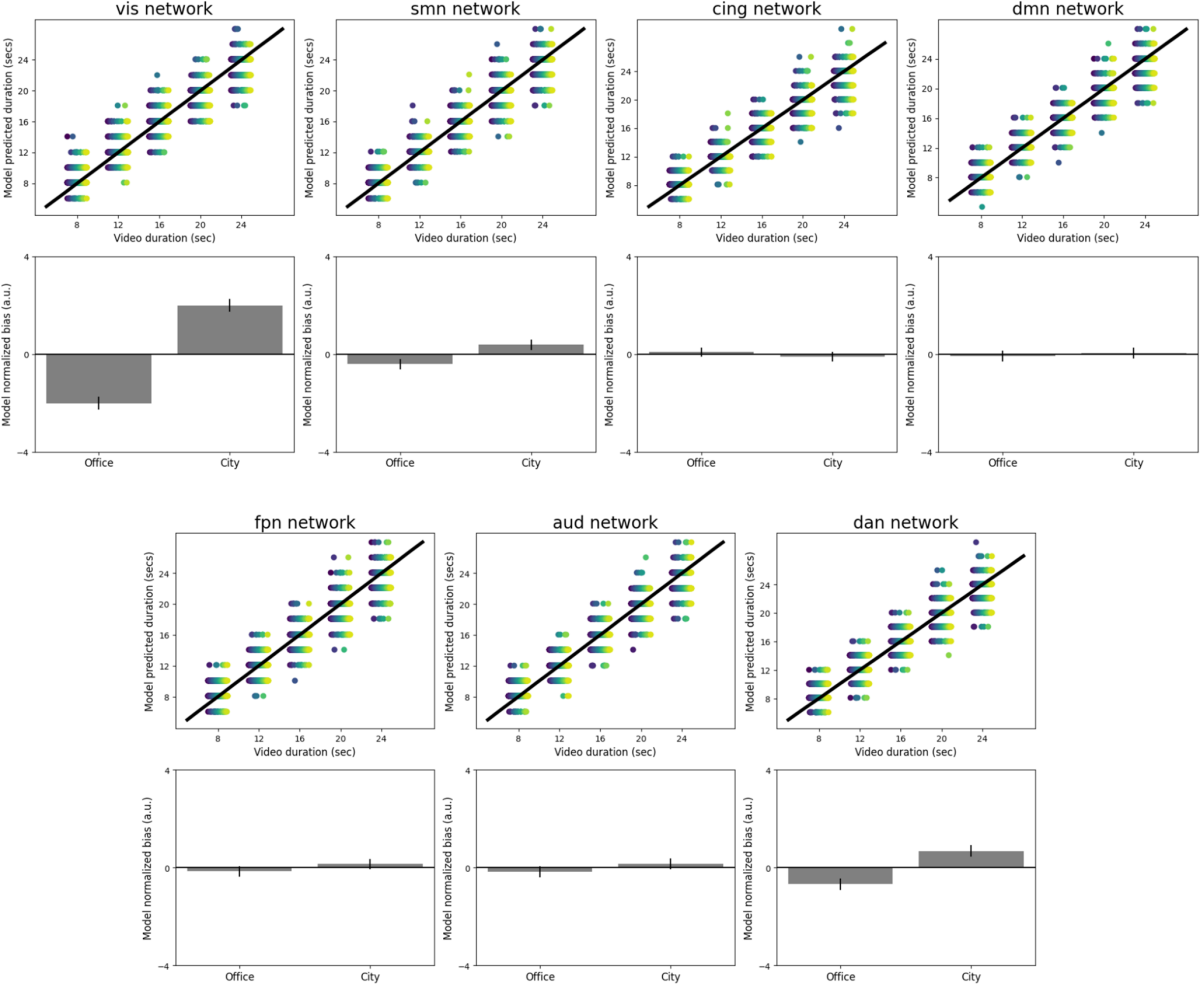
Model predictions for each functional network: (Above) Association between the video duration and model-predicted duration obtained from the model of each functional network. Different colors of points represent different participants and each point is data from one trial. (Below) Average normalized bias estimated by the model depending on the type of video for the models of each functional network. Error bars represent +/- SEM across participants. FPN – Frontoparietal Network, DAN – Dorsal Attention Network, CON – Cingulo-Opercular Network, DMN – Default Mode Network, VIS – Visual Network, AUD – Auditory Network, SMN – Sensorimotor Network. Source: de Souza et al., 2024

Table 1 presents the effect of video type on normalized bias observed for each functional brain network evaluated when considering the pre-registered parameters for Eq. 3. The only models that showed significant results regarding the association between model and human normalized biases were those of the visual network and the dorsal attention network, considering the significance level of 0.05 for the chi-squared test (critical value = 7.879). Figure 1 shows that the visual, somatomotor and dorsal attention models reproduce, to varying degrees, the average behavior of under- and over-estimation of office and city videos, respectively.

**Table 1:** Effect of video type measured with the chi-squared test upon the linear mixed models using data from different networks. FPN – Frontoparietal Network, DAN – Dorsal Attention Network, CON – Cingulo-Opercular Network, DMN – Default Mode Network, VIS – Visual Network, AUD – Auditory Network, SMN – Sensorimotor Network

Network	Bias difference	95% CI	Chi-squared	p-value
vis	4.0 ± 0.53	[2.96, 5.04]	72.77	0.00
smn	0.8 ± 0.44	[-0.06, 1.66]	3.52	0.061
cing	-0.19 ± 0.39	[-0.95, 0.57]	0.67	0.413
dmn	0.12 ±0.44	[-0.74, 0.98]	0.28	0.597
fpn	0.29 ±0.42	[-0.53, 1.11]	0.67	0.413
aud	0.33 ±0.46	[-0.57, 1.23]	0.45	0.502
dan	1.36 ± 0.48	[0.42, 2.3]	8.98	0.003

**Fig. 2:**
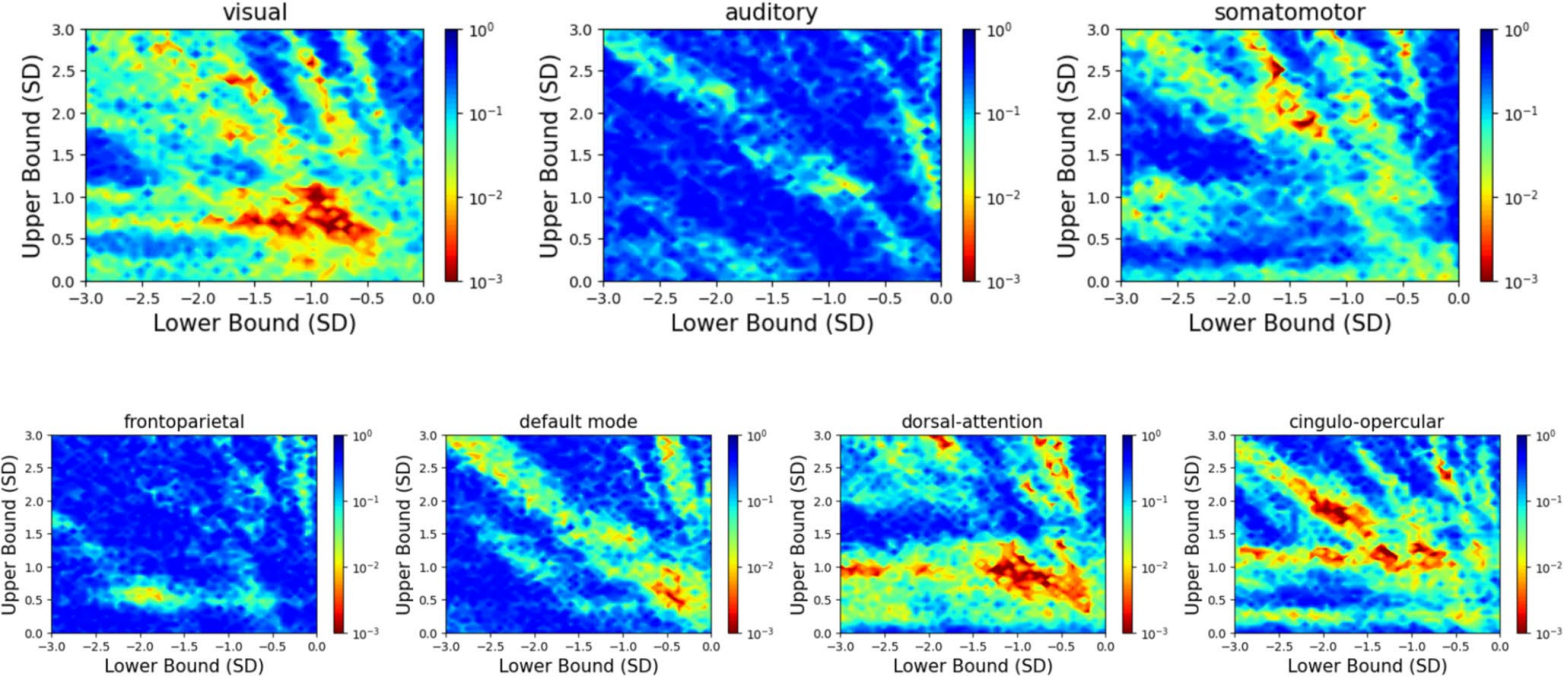
Robustness analysis upon each functional network model. Heat map depicting p-values for the association between human bias and model bias, as a function of minimum (x-axis) and maximum (y-axis) criterion values. Dark colors represent regions where the association was non-significant at α_0.05_ or negative. Source: de Souza et al., 2024

**Fig. 3:**
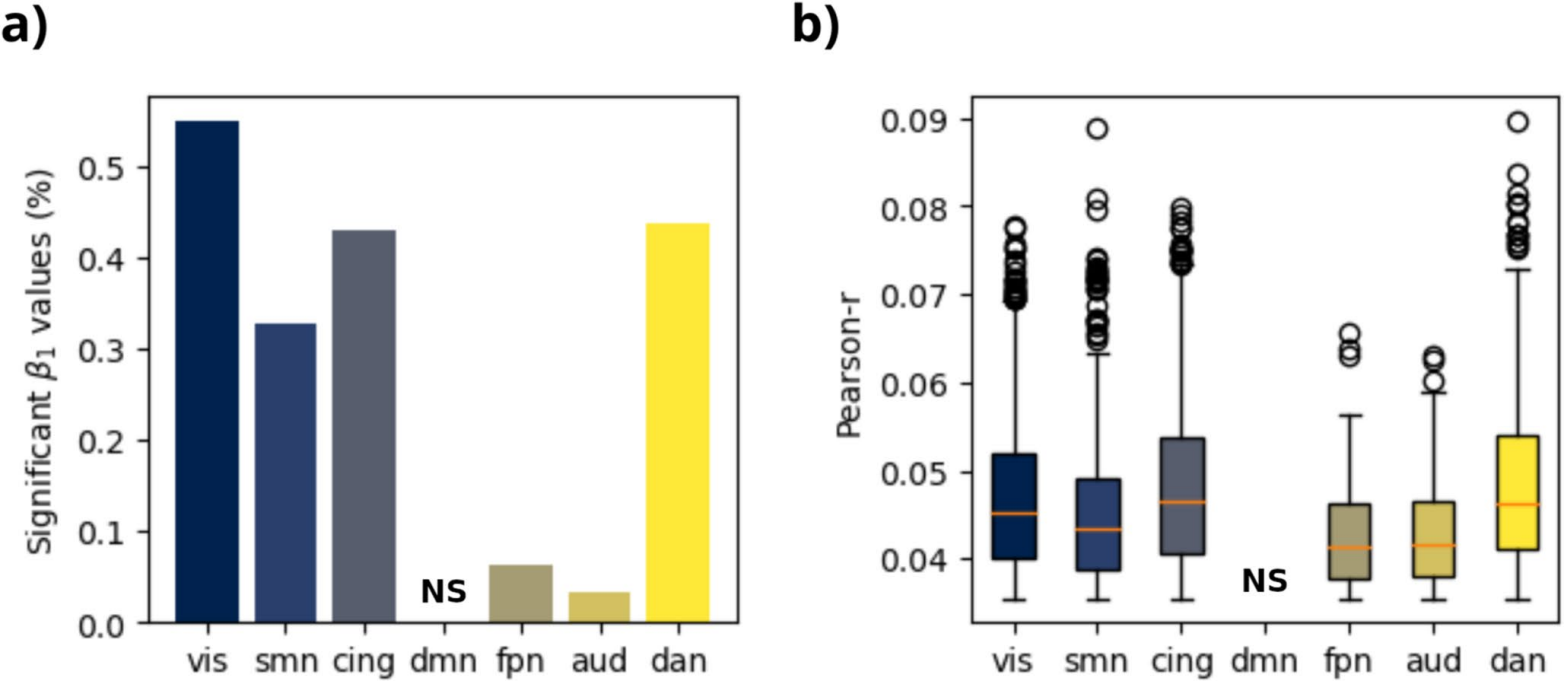
Significant associations between human and model bias across functional networks. (**a**) Fraction of significant linear regression β1 values (*p* < 0.05) across a wide range of criterion function parameters. (**b**) Box-plot of the linear correlation (Pearson-r) between human and model bias predicted from criterion parameters that implied significant β1 values. FPN – Frontoparietal Network, DAN – Dorsal Attention Network, CON – Cingulo-Opercular Network, DMN – Default Mode Network, VIS – Visual Network, AUD – Auditory Network, SMN – Sensorimotor Network. Source: de Souza et al., 2024

Within the functional atlas, we observed that four models showed consistent positive correlations between normalized bias and salient events across criterion parameter values: visual, dorsal-attention, cingulo-opercular and somatomotor networks (Figs. 2 and 3). Fronto-parietal and auditory networks showed no association, while the default-mode network model presented probably spurious or artefactual associations in small and localized sets of parameter values. Importantly, positive correlations between human and model bias were always weak across functional networks (Fig. 3).

### Approach 2: prediction of normalized bias from bold time-series

We could not predict normalized bias with the LSTM model for any brain parcellation choice across different combinations of parameters (Figure S1). Also, this model was not able to discriminate between video types; the prediction performances fluctuated around 0.5 area under the curve (AUC) score, which means a performance equivalent to chance. Training the model with unsmoothed fMRI data did not improve the prediction performance (results not shown).

When attempting to predict the video duration values, we obtained positive correlations for different combinations of training parameters (Figure S2). However, it is a trivial result since time-series size and video duration are intrinsically associated. We used the parameter combination that yielded the best performance to calculate a model-related bias using Eq. 1. We could not find an association between model bias and human bias using this approach.

### Approach 3: prediction of normalized bias from functional connectivity

**Fig. 4:**
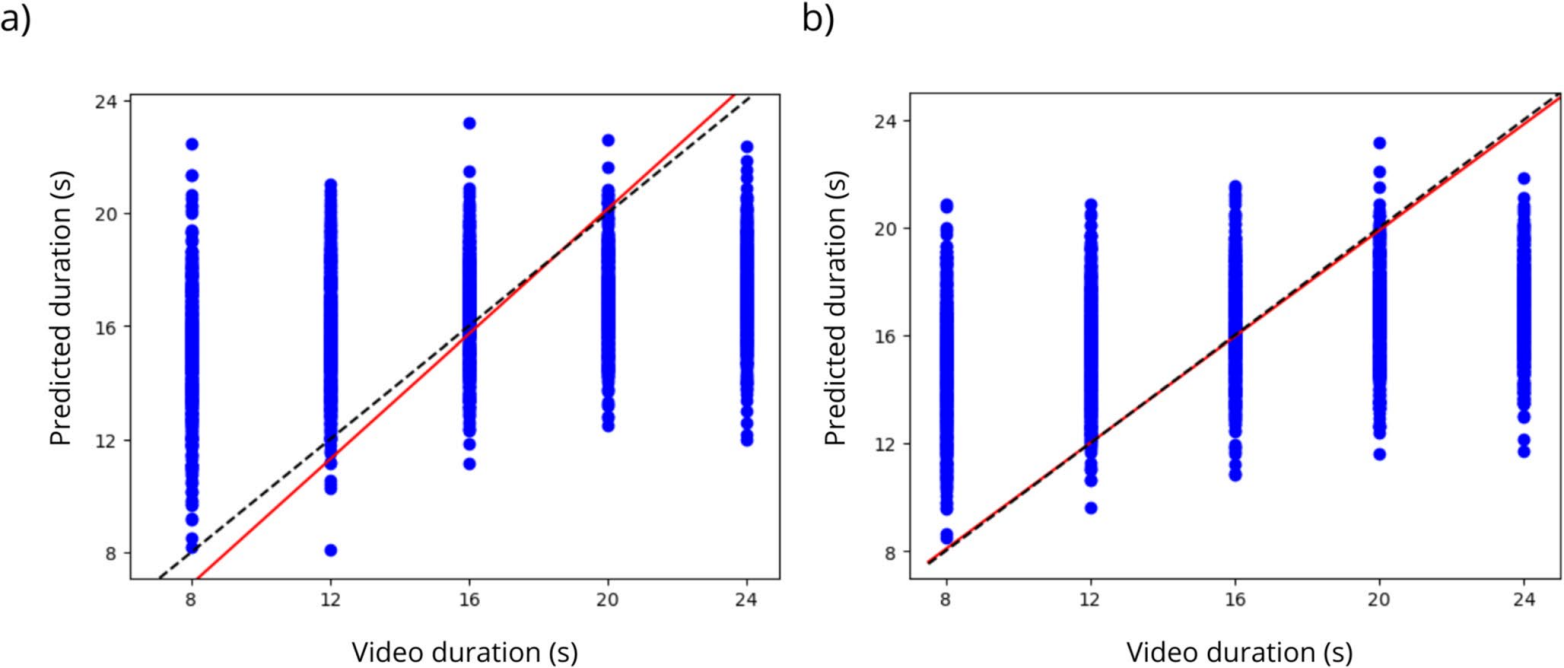
(**a**) Performance of the Elastic-net model on the prediction of trial-by-trial video duration from whole-brain functional connectivity. The dashed line represents the identity line and the red line is the linear regression. (**b**) Prediction performance when using data from random sampling of the subject full block time-series. Source: de Souza et al., 2024

Using the elastic-net model, we could not capture any association between FC and normalized bias. Video duration can be predicted by FC extracted from the whole-brain functional atlas (Fig. 4a) with moderate performance (R^2^ = 15,5%). To determine whether this correlation was independent of the size of the time-series, we adjusted the model using randomly sampled time-series from each subject’s fMRI block to compute trial-by-trial FC values. In Fig. 4b, we show that the prediction performance with random data (R^2^ = 13,8%) was very close to that obtained with task-related data. Furthermore, we could not find an association between human bias and the normalized bias reconstructed from predicted video duration.

## Discussion

### Approach 1: prediction of normalized bias from salient events

By using an atlas that categorizes brain regions into functional networks, we found evidence that the dorsal attention (DAN), the cingulo-opercular (CON) and the somatomotor (SMN) networks, in addition to the expected visual network, are relevant for the human subjective perception of time (Figs. 2 and 3). The DAN is closely linked to spatial attention functions, such as directing eye movements toward specific visual stimuli and/or maintaining attention on these stimuli over time (Kincade et al., 2005). The DAN includes the inferior parietal lobule (IPL), a region commonly associated with time perception research. Specifically, the right IPL is more linked to quantifying durations (Bueti et al., 2008; Jantzen et al., 2005), while the left IPL is associated with predicting rhythms (Ross et al., 2018). A meta-analysis by Naghibi et al. (2024) found that tasks involving visual and single-interval timing selectively activate regions in the right frontoparietal network, which includes the MFG and the IPL.

The salient events from the cingulo-opercular network (CON) also yielded consistent correlations between human and model bias (Fig. 2); however, the predictions obtained from the CON data could not reproduce the effect of video type on normalized bias, when applying the pre-registered criterion values (Fig. 1). As a task control network (Sestieri et al., 2014), the CON is involved in a wide range of cognitive processes. The insula and the IFG are contained in the CON and were reported in the literature as associated with timing of durations. In addition to the pre-SMA, the insula has been identified as a key brain region involved in duration processing across various timing contexts (Naghibi et al., 2024), it is considered a central hub for interoceptive processes and has been proposed as a potential neural substrate for temporal awareness (Craig, 2009) due to its role in the detection of salient stimuli.

Furthermore, the SMN model also presented consistent associations to human bias (Fig. 3). Sensorimotor regions have been described as relevant to time perception across several contexts, including the timing of visual stimuli (Naghibi et al., 2024; Nani et al., 2019), the most prominent and almost ubiquitous structure is the pre-SMA. Actually, Naghibi et al. (2024) highlight that sensorimotor processes have a crucial role in the abstract representation of time. This association may be emphasized by the nature of the presented stimulus (e.g. walking in the city), since the SMN is related to motor imagery processes (Zhang et al., 2014).

In this work, we only investigated these networks as large average regions of interest; future work may evaluate more precise associations of specific regions contained in these networks with subjective time and replicate the same analytical methodology using alternative task-fMRI stimuli categories (e.g. auditory and tactile stimulus). Importantly, salient event detection is inherently constrained by the temporal resolution of the fMRI acquisition, such that rapid neural changes occurring at time scales shorter than this sampling interval may be temporally blurred or not captured by the frame-to-frame BOLD differences used in this analysis. Moreover, Approach 1 is inherently flexible and can be readily extended to alternative brain atlases, including hybrid cortical–subcortical parcellation schemes. This would allow future investigations to explicitly incorporate subcortical structures—such as those previously implicated in interval timing—while preserving the same analytical framework adopted in the present study.

Finally, a limitation of the present study is the absence of explicit subjective saliency ratings. While saliency was operationalized implicitly through stimulus-driven neural dynamics, subjective reports could provide complementary insight into how individual experience, attention, and interpretation interact with the physical properties of the stimuli. Future studies combining neural measures with explicit behavioral assessments of saliency may help to further disentangle the respective contributions of perceptual complexity and subjective experience to time perception.

### Approach 2: prediction of normalized bias from bold time-series

As far as we know, this is the first reported work trying to map the direct association between brain fMRI time-series and subjective time. Importantly, the salient events calculated in the first approach are also derived from the pre-processed fMRI series; however, the difference in this approach is that we attempted to capture relevant features without a pre-defined criterion function to classify salient events. Using a deep recurrent model, we did not observe evidence for this association when using data from a whole-brain cortical atlas or from the visual cortex or the visual functional network (Figure S1). The association between the time-series data and the video duration is trivial and is not related to subjective time since these variables are positively correlated. Furthermore, the reconstructed model-related bias is also not correlated to human bias, thus, our model is not able to capture salient changes or any other intrinsic dynamical pattern from BOLD time-series that may be associated with normalized bias.

The most likely explanation for the model flaw is that there is no direct association between the evaluated variables, but some limitations from our approach might have mitigated desirable results. The main limitation is the dataset size, since the dataset used here contains data from only 38 subjects. We assumed that using each subject trial data as a separate sample could be sufficient for the model to track the effect of variations in brain dynamics on the variations of subjective bias, however, this assumption might be wrong. Future work may investigate if a larger dataset would improve model performance. Another potential limitation was the use of a group-based parcellation derived from a different MRI dataset (HCP), which may introduce inaccuracies in the definition of brain regions of interest.

### Approach 3: prediction of normalized bias from functional connectivity

As far as we know, this is the first work trying to evaluate the association between whole-brain functional connectivity and subjective time metrics. Here, using a regularized linear regression model, we could not find evidence for this association (Figure S1). Although the most likely explanation is that there is no direct association, the limitations described for approach 2 are also valid for this approach. Also, the functional atlas investigated in this work contemplates only cortical regions; it has been shown in the literature that subcortical regions and the neural communication between cortical and subcortical regions may play an important role in time perception (Naghibi et al., 2024; Liu et al., 2023). Although different regions appear to be involved in temporal estimation, the interaction between these regions in the form of functional connectivity calculated as the Pearson correlation coefficient did not capture this, not even within the visual network. Future work should explore if the addition of subcortical related connectivity would improve model performance.

## Conclusions and limitations

An influential recent framework for understanding the neural basis of time perception proposes that subjective time is determined by the accumulation of salient changes in perceptual processing hierarchies. This hypothesis was first tested by Sherman et al. (2022), who employed an approach combining BOLD fMRI with visual stimulation. Their main finding suggests that subjective time—measured as a normalized duration bias in the estimation of silent videos—is linked to changes in the neural activity within the visual cortical hierarchy, rather than in non-related control hierarchies such as the auditory or somatosensory systems.

In this study, we sought to extend the existing framework by examining how salient changes across different brain resting-state networks relate to subjective time. In addition to the visual network—which was expected to be associated with normalized bias—we found that the dorsal attention, cingulo-opercular, and somatomotor networks also showed positive correlations between human and model-predicted biases. These results highlight that temporal estimation involves not only stimulus-driven sensory networks but also broader functional systems. Nevertheless, our approach is anatomically coarse and does not capture the functional contributions of specific regions within these networks. These findings open new avenues for investigating the distinct roles of these networks in time perception, particularly in the context of prospective duration estimation.

As a second novel approach, we explored whether it would be possible to directly extract dynamic information from the visual cortex or whole-brain BOLD time-series that relates to subjective time—without relying on predefined criteria to detect salient changes. Using a deep recurrent neural network, we were unable to find positive correlations between trial-by-trial model predictions and human biases in any of the tested conditions. Given the use of a deep learning framework, it is likely that the dataset was not large enough for the model to reliably capture temporal processing–related information.

As a follow-up to our findings that broader resting-state networks beyond the visual cortex are involved in temporal estimation, we sought to examine whether specific brain regions show functional connectivity patterns associated with subjective time. To this end, we trained a regularized linear model to predict normalized duration bias from functional connectivity values, using both whole-brain and visual network–restricted connectivity profiles. However, this approach did not yield significant results.

In summary, this study investigated the relationship between subjective time and various features derived from BOLD fMRI data across multiple cortical networks and processing hierarchies. Our findings highlight the contribution of additional functional networks—beyond the visual system—to interval timing, particularly in the context of salient perceptual events. One potential limitation is our reliance on broad network definitions and large-scale processing hierarchies, which may have obscured finer-grained correlations. Future research should explore whether incorporating functional connectivity between specific regions previously linked to time perception could enhance model performance. Additionally, our analyses were constrained by the use of previously acquired data from Sherman et al. (2022), which was collected using protocols tailored to a different set of hypotheses. This inherently limits the flexibility for implementing and testing new analytical approaches.

## Supplementary Information

Below is the link to the electronic supplementary material.

Supplementary Material 1

## Data Availability

The dataset used in this study was provided by Sherman et al. (2022) and is available for download at the OSF repository https://osf.io/2zqfu.

## Code availability

All analysis scripts are available at https://github.com/inbrainlab/time_perception_fMRI.
