# Supplementary material for: Brain regions beyond the visual cortex are relevant to subjective time prediction from fMRI salient events in a visual naturalistic context
Source: Brain Imaging Behav. 2026 Mar 21;20(2):58. doi: 10.1007/s11682-026-01128-8 (PMC13005859; doi:10.1007/s11682-026-01128-8)
Supplement: Supplementary file 1 — Supplementary Material 1 [file 11682_2026_1128_MOESM1_ESM.docx]

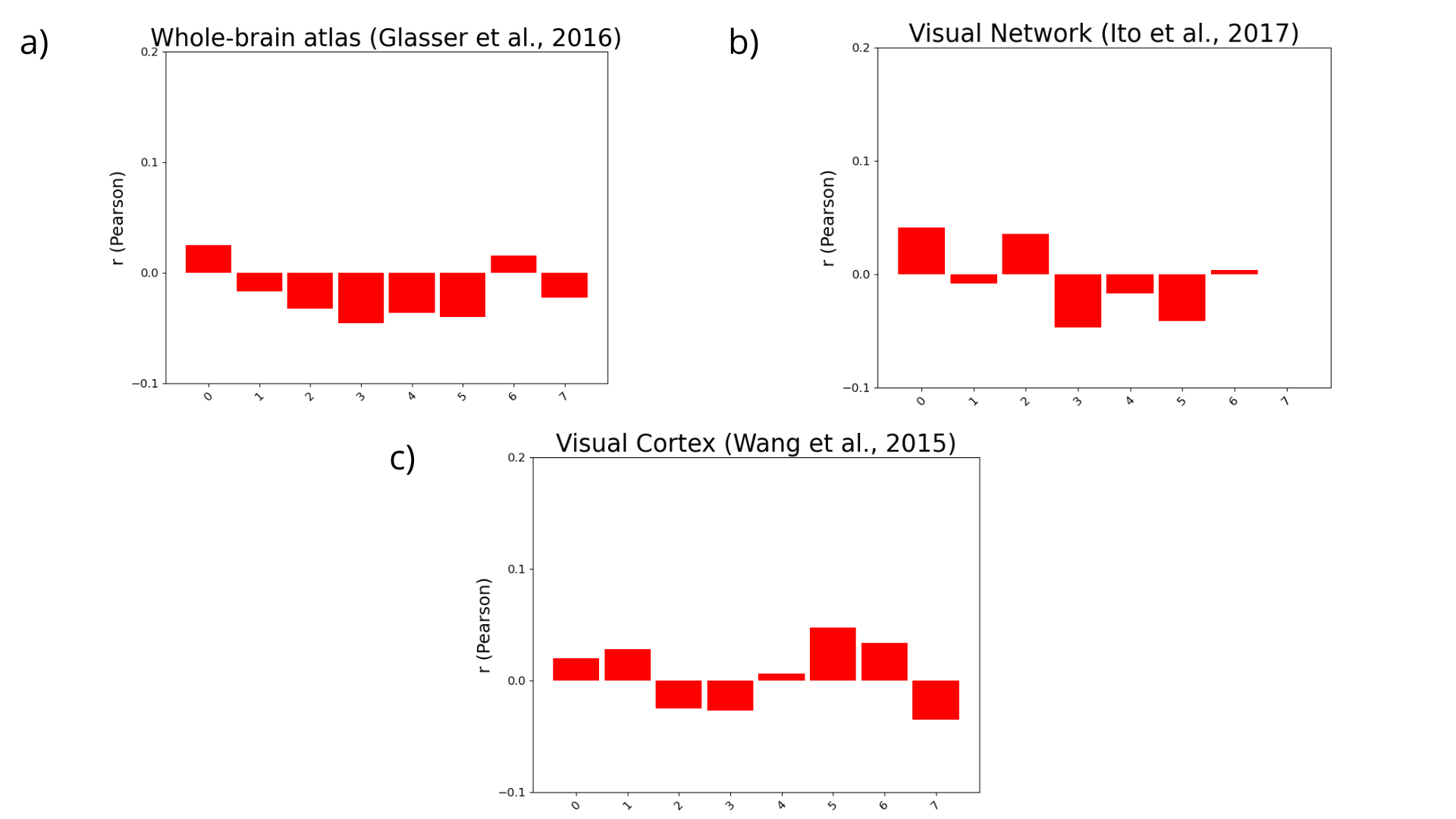


Figure S1. Pearson correlation coefficient (r) values between actual and predicted normalized bias across several LSTM model hyperparameter (learning rate, scheduler step size and scheduler gamma value) combinations for each evaluated brain parcellation.


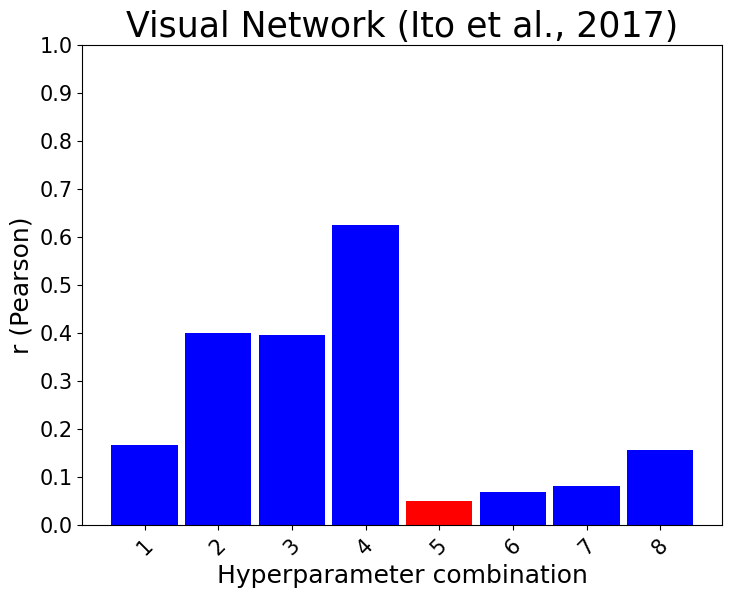


Figure S2. Pearson correlation coefficient (r) values between actual and predicted video duration values across several LSTM model hyperparameter (learning rate, scheduler step size and scheduler gamma value) combinations within the brain visual network.
